# Editorial: Tumor Microenvironment and Cancer Cell Interactions in Solid Tumor Growth and Therapy Resistance

**DOI:** 10.3389/fcell.2022.896194

**Published:** 2022-04-08

**Authors:** Maria Rosaria Ruocco, Annalisa Lamberti, María José Serrano, Giuseppe Fiume, Alessandro Arcucci

**Affiliations:** ^1^ Department of Molecular Medicine and Medical Biotechnology, University of Naples Federico II, Naples, Italy; ^2^ Integral Oncology Division, Virgen de las Nieves University Hospital, Granada, Spain; ^3^ GENYO, Centre for Genomics and Oncological Research: Pfizer/University of Granada/Andalusian Regional Government, Granada, Spain; ^4^ Instituto Biosanitario Granada (iBS-Granada), Granada, Spain; ^5^ Department of Experimental and Clinical Medicine, University of Catanzaro “Magna Graecia”, Catanzaro, Italy; ^6^ Department of Public Health, University of Naples Federico II, Naples, Italy

**Keywords:** tumor microenviroment, cutaneous melanoma, tumour extracellular matrix, therapy resistance, cancer immune response

Solid tumour tissues contain a tumour microenvironment (TME) which influences tumour progression and therapy resistance (Romano et al., 2021). TME is a network including non-cancer stromal cells, extracellular matrix (ECM), growth factors, nutrients, blood and lymphatic vessels (Avagliano et al., 2020b), and its structure and components, depending on the type and location of the tumour, make each solid tumour unique (Granato et al., 2017; Avagliano et al., 2020a). Moreover, the TME plasticity leads to its evolution with disease and adaptation to cancer cell and environmental alterations. The components of TME by interacting with each other and with tumour cells generate a cancer niche that sustains immunosuppressive processes, drug resistance, cancer recurrence and dissemination that represent the main causes of cancer related deaths.

**FIGURE 1:**
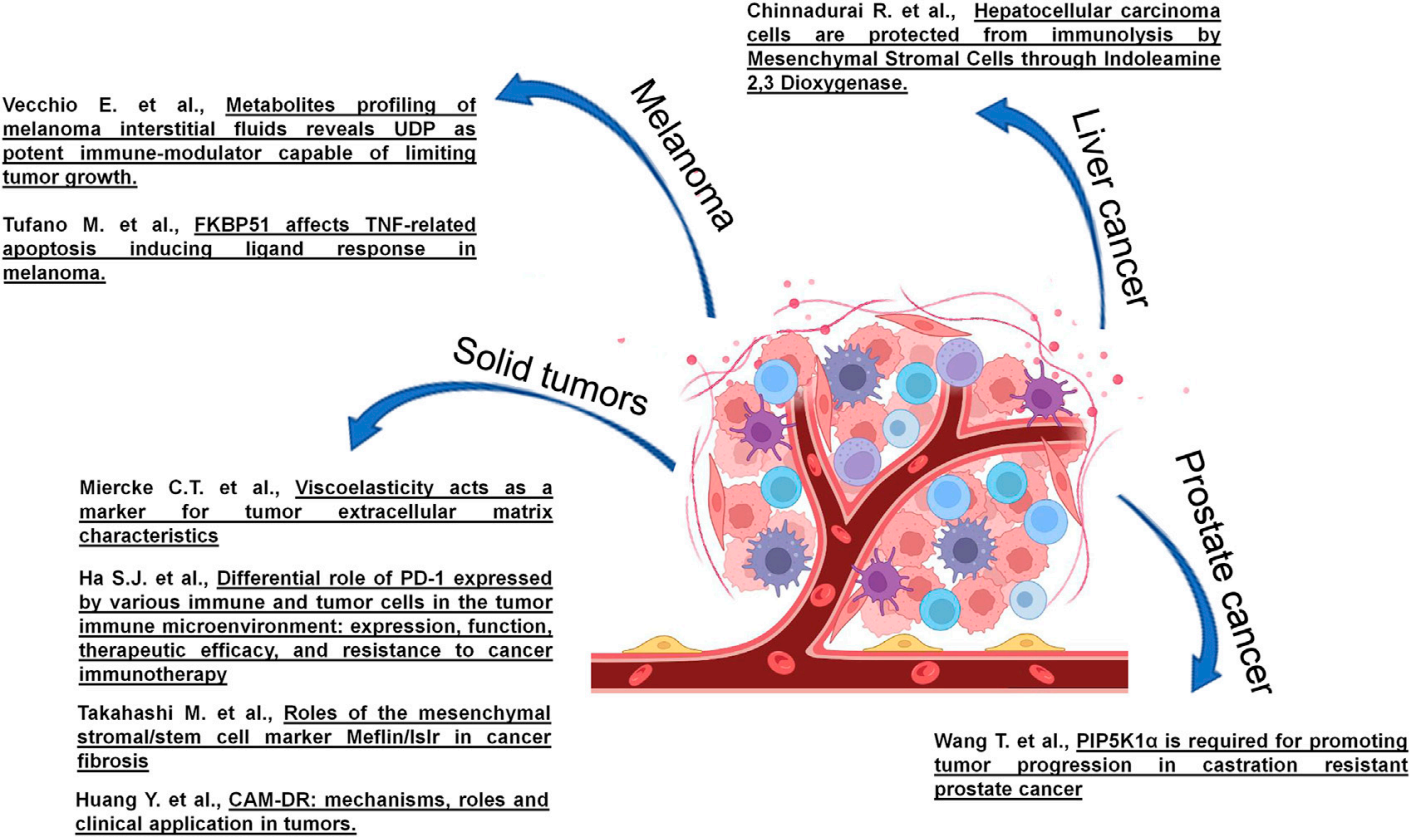
Studies on TME of different cancers published in the research topic entitled “Tumor Microenvironment and Cancer Cell Interactions in Solid Tumor Growth and Therapy Resistance.

This research topic includes eight articles, four reviews and four original articles (Figure 1). Some articles describe the role of extracellular matrix in tumour progression, metastasis formation and resistance to cancer therapy. Other articles describe both the effects of specific proteins in modulating growth, survival and invasion capability of cancer cells as well as the cross-talk between immune, stromal and tumour cells, mediated by cytokines, chemokines or metabolites, capable of creating immunosuppressive or immunostimulatory environments, in different tumour contexts.

More specifically, Huang et al. exhaustively described in their review the interactions between tumour cells and extracellular matrix components, including both filamentous proteins (laminin, fibronectin, and collagen) and paracrine factors (cytokines, chemokines, growth factors, exosomes), involved together in the process of cell adhesion-mediated resistance (CAM-DR), essential in different tumours including multiple myeloma, non-Hodgkin’s lymphoma, leukaemia and solid tumours. Finally, they focused on the therapeutic drugs and strategies that could reverse or inhibit CAM-DR process.

Takahashi et al., in their review, after an introduction on the impact of fibroblasts in extracellular matrix remodelling, described the role of Meflin/Islr, a GPI-anchored cell surface protein, a novel marker of Mesenchymal Stromal/Stem Cells (MSC) and cancer associated fibroblasts (CAFs) in different tumours, including pancreatic and colorectal cancers, and in fibrosis of some tissues.

Claudia Tanja Mierke provided us an interesting point of view on the particular importance of a chemical-physical property of extracellular matrix, named viscoelasticity that is referred to a material that exhibits characteristics of both an elastic solid and a fluid, in cancers. Therefore, an accurate measurement of the mechanical features of the tumour extracellular matrix through several techniques could represent a novel marker for prognosis and diagnosis of cancers and provide a further level of comprehension of biological processes in cancer pathogenesis.

Ha et al. exhaustively reviewed different role of PD-1, based on its expression in distinct tumour-infiltrating immune subsets. In particular, they focused on how cell-type-specific ablation or blockade of PD-1 influences tumor growth in a murine tumor model. Further, the authors also describe the effects of PD-1 blockade on TI immune cells in human cancer patients.

Tufano et al., in their article, described a novel pro-oncogenic function of FKBP51 protein in melanoma, which supports NF-κB-mediated resistance and cancer stemness/invasion epigenetic programs. It is worth mentioning that an aberrant expression or activity of NF-κB family member proteins is a common hallmark of various cancers. The NF-κB proteins are a family of transcription factors that regulate the expression of a huge amount of genes and modulate a wide range of cellular processes affecting carcinogenesis, including protein synthesis (Fiume et al., 2013; Pisano et al., 2015), viral expression (Puca et al., 2007; Vitagliano et al., 2011), immune cell development (Fiume et al., 2013; Albano et al., 2019), inflammation (Taniguchi and Karin, 2018), apoptosis (Albensi) and cell survival (Luo et al., 2005), autophagy (Trocoli and Djavaheri-Mergny, 2011), cell motility (Chang et al., 2021). In particular, they found that FKBP51 depletion caused an increased expression of TNF-related apoptosis-inducing ligand (TRAIL)-R2 (DR5), inducing a sensitization of melanoma cells to TRAIL-dependent apoptosis. Their results suggest a novel therapeutic strategy for melanoma treatment.

Chinnadurai et al. described an interesting cross-talk between cells in a tripartite tumour model consisting of PBMCs, MSCs and hepatocellular carcinoma cell line (HepG2). They showed that in co-culture of HepG2 cells and activated PBMCs, HepG2 cells undergo PBMC mediated cytolysis while MSCs protect HepG2 cells from PBMC mediated lysis, through an IDO (indoleamine 2,3 dioxygenase)-dependent mechanism. Finally, they observed that the blockade of IDO activity completely abolishes the ability of MSCs to protect HepG2 cells from cytolysis induced by PBMCs, providing a novel immunotherapeutic strategy for hepatocellular carcinoma treatment.

Vecchio et al. developed a metabolomic technique to analyse the metabolite composition of melanoma interstitial fluid and compare it to plasma of mice engrafted or not with melanoma cells. Among the most enriched metabolites within tumour interstitial fluid, they found guanosine diphosphate (GDP) and uridine diphosphate (UDP). These metabolites acted as stimulator of immune response, increasing the percentage of CD4^+^CD25^+^FoxP3^–^, inducing the phosphorylation of ERK, STAT1, and STAT3 and stimulating the activity of NF-κB subunits p65, p50, RelB, and p52. Further, they observed an increased expression of Th1/Th17 markers and a reduced expression of IL13, a Th2 marker. Finally, they observed that intra-tumoral administration of UDP in mice reduced tumour growth and necrotic areas associated to a higher presence of MHCII^hi^ tumour-associated macrophage (TAM) and of CD3^+^CD8^+^ and CD3^+^CD4^+^ tumour-infiltrating T-lymphocytes (TILs). Therefore, they found a novel mediator of immune response, which could potentially represent an adjuvant in cancer immunotherapy.

Wang T. et al. show that the depletion or blockade of PIP5K1α protein suppress growth and invasion of castration-resistant prostate cancer cells (CRPC). The same results were obtained deleting the N-terminal domain of PIP5K1α, required for regulation of mRNA expression and protein stability, in CRPC cells. From a mechanistic standpoint, PIP5K1α acts as an upstream regulator of the androgen receptor (AR) and modulates the expression of AR target genes CDK1 and MMP9. Therefore, their studies identify a novel protein that represents an intriguing target for cancer treatment.

Taken together, these studies on processes modulating the interactions within cancer niche of solid tumours represent one of the most important areas of oncological research that could lead to significant therapeutic advances in medical oncology.

However, we believe that studies focusing on the reversibility of phenotype of TME components that affect cancer development, dissemination, therapeutic resistance and immune escape are missing in this Research Topic and are under-represented in the literature on TME. Understanding whether all TME components are irreversibly differentiated into a pro-tumorigenic phenotype or whether some components of TME can go back to physiological phenotype, acting as anti-tumour components, should be of great relevance.

The identification of molecules and signalling pathways regulating the plasticity of both normal and tumour microenvironment could allow the development of new cancer therapies capable of reducing side effects, improving significantly disease outcome and reduce therapy resistance.
